# LINC00511 promotes melanoma progression by targeting miR-610/NUCB2

**DOI:** 10.1515/med-2023-0628

**Published:** 2023-03-01

**Authors:** Guangjing Zhang, Zhengxiang Wang, Jie Liu, Shijun Feng, Shanshan Ji, Dongfang Ai

**Affiliations:** Department of Dermatology, Hebei Province Cangzhou Central Hospital, No. 16, Xinhua West Road, Cangzhou, Hebei, 061001, China; Department of Dermatology, Hebei Province Cangzhou Central Hospital, Hebei, 061001, China

**Keywords:** LINC00511, melanoma, proliferation, metastasis, nucleobindin-2

## Abstract

Long intergenic noncoding RNA 00511 (LINC00511) predicts poor prognosis in various malignancies and functions as an oncogene in distinct malignant tumors. The role of LINC00511 in melanoma progression was assessed. In our research, expression of LINC00511 in melanoma cells was detected by quantitative reverse transcription PCR. Colony formation and CCK8 assays were used to detect cell proliferation. Cell metastasis was evaluated by transwell and wound healing assays. Downstream target of LINC00511 was investigated by luciferase activity assay. As a results, LINC00511 was elevated in melanoma cells and tissues. Loss of LINC00511 decreased cell viability, reduced proliferation, invasion, and migration of melanoma. miR-610 was target of LINC00511, and miR-610 binds to 3′UTR of nucleobindin-2 (NUCB2). Inhibition of miR-610 attenuated LINC00511 deficiency-induced decrease of NUCB2 in melanoma cells. Loss of miR-610 weakened LINC00511 deficiency-induced decrease of cell viability, proliferation, invasion, and migration of melanoma. In conclusion, silence of LINC00511 reduced cell proliferation and metastasis of melanoma through down-regulation of miR-610-mediated NUCB2.

## 1 Introduction

Melanoma is a deadly form of skin cancer [1]. However, melanoma has the lowest incidence among all skin cancers, melanoma, as the most malignant and aggressive type of skin cancer, accounts for 75% of skin cancer-related deaths [2]. Moreover, elucidation of the pathogenesis involved in melanoma, as well as targeted therapy and immunotherapy, has been made great advancement; the prognosis of patients with melanoma is still not optimistic [2]. Therefore, novel prognostic biomarkers and therapeutic targets are essential for the improvement of unsatisfactory prognosis in melanoma patients.

Long non-coding RNAs (LncRNAs) were widely known as prognostic biomarkers of tumors [3]. Emerging evidence has shown that lncRNAs were also implicated in proliferation and metastasis of melanoma, and lncRNAs were involved in drug resistance of melanoma [4]. LINC00511 was shown to be associated with metastasis and poor prognosis in malignant tumors and predicted poor disease-free survival and overall survival [5]. Moreover, LINC00511 functioned as an oncogene in non-small cell lung cancer [6], colon cancer [7], glioma [8], breast cancer [9], gastric cancer [10], and cervical cancer [11]. The silence of LINC00511-promoted cell apoptosis and inhibited the proliferation of bladder cancer [12]. LINC00511 was identified as a splicing factor, proline- and glutamine-rich-enriched lncRNA, and promoted glycolysis of melanoma cells [13]. However, the role of LINC00511 in the metastasis of melanoma remains unclear.

Generally, lncRNAs function as miRNA sponges, regulate downstream target genes and construct a competing endogenous RNA network to mediate melanoma progression [14]. For example, LINC00511 binds to miR-625-5p and accelerates proliferation and metastasis of gastric cancer [15]. Depletion of LINC00511 increased miR-625-5p expression and decreased pyruvate kinase M2 to suppress the glycolysis of melanoma cells [13]. The miR-610 exerted a tumor suppressive effect on melanoma through downregulation of LDL receptor-related protein 6 [16]. LINC00511 was predicted to bind to miR-610. Therefore, LINC00511 might contribute to melanoma progression through downregulation of miR-610.

In this study, the effects of LINC00511 on cell proliferation, invasion, migration, and epithelial–mesenchymal transition of melanoma cells were investigated. Downstream miRNA-mRNA network involved in LINC00511-mediated melanoma progression might provide a potential target for melanoma.

## 2 Materials and methods

### 2.1 Bioinformatic analysis

GEPIA (http://gepia.cancer-pku.cn/) was used to analyze the expression of LINC00511 in skin cutaneous melanoma tissues.

### 2.2 Cell culture and treatment

Human epidermal melanocytes (HEMa-LP) and melanoma cells (A375, A-2058, SK-MEL-28, MV3) were acquired from ScienCell Research Laboratories, inc. (Carlsbad, CA, USA). Cells were grown in RPMI 1640 medium containing 10% fetal bovine serum (Gibco, Grand Island, NY) and incubated in a 37°C humidified incubator. The siRNAs targeting LINC00511 (si LINC00511#1 and si LINC00511#2), pcDNA-LINC00511, miR-610 mimic, miR-610 inhibitor, and the negative controls were purchased from GenePharma (Shanghai, China). A375 and SK-MEL-28 were transfected with siRNAs, pcDNA vectors, mimics, or inhibitors using Lipofectamine 2000 (Invitrogen, Carlsbad, CA, USA).

### 2.3 qRT-PCR

Cells were lysed in Trizol (Sigma-Aldrich, St. Louis, MO, USA) or miRcute miRNA isolation kit (Tiangen, Beijing, China). Isolated RNAs were reverse-transcribed into cDNAs, and the cDNAs were subjected to qRT-PCR analysis using SYBR Green Master (Roche, Mannheim, Germany). Expression of LINC00511 (Forward: 5′-TGGCTTGTCTTCCATCGTCC-3′ and Reverse: 5′-GCACGAGGGTTGTTACAGGA-3′), miR-610 (Forward: 5′-CGCGGATCCGGGGCAACACTTAACATA-3′ and Reverse: 5′-CCGCTCGAGTTGGGATCTGGTGTTTATT-3′), and NUCB2 (Forward: 5′-AAAGAAGAGCTACAACGTCA-3′ and Reverse: 5′-GTGGCTCAAACTTCAATTC-3′) were determined by 2^−△△CT^ method. GAPDH (Forward: 5′-GGATTTGGTCGTATTGGG-3′ and Reverse: 5′-GGAAGATGGTGATGGGATT-3′) and U6 (Forward: 5′-CTCGCTTCGGCAGCACA-3′ and Reverse: 5′-AACGCTTCACGAATTTGCGT-3′) were used as endogenous controls.

### 2.4 Cell viability assay

A375 and SK-MEL-28 were seeded in 96-well plates and subjected to different transfections for 24 h. Cells were then cultured in RPMI 1640 medium for 24, 48, or 72 h. Cells were treated with CCK8 solution (Beyotime, Beijing, China) for 2 h, and absorbance at 450 nm was examined via Microplate Autoreader (Thermo Fisher, Waltham, MA, USA).

### 2.5 Cell proliferation assays

A375 and SK-MEL-28 were seeded in six-well plates and subjected to different transfections. Cells were cultured in RPMI 1640 medium for 10 days. Cell colonies were fixed in methanol and stained with crystal violet and then photographed under a light microscope (Olympus, Tokyo, Japan).

### 2.6 Wound healing assay

A375 and SK-MEL-28 were seeded in six-well plates and subjected to different transfections. The middle of the plates was scratched by a pipette tip. Cells were observed under the microscope 24 h later, and the wound width was calculated using ImageJ software.

### 2.7 Transwell assay

A375 and SK-MEL-28 were subjected to different transfections and suspended in serum-free RPMI 1640 medium. Cells were plated in the upper chamber of a Matrigel-coated well (Corning, Tewksbury, MA, USA). RPMI 1640 medium containing 20% fetal bovine serum was filled in the lower chamber. Cells in the lower chamber were fixed in methanol and stained with crystal violet 24 h later. Cells were observed under a microscope. The number of invasive cells was calculated by ImageJ software.

### 2.8 Dual-luciferase reporter assay

Sequences of wildtype or mutant 3′-UTR of LINC00511 or NUCB2 were constructed into pmirGLO luciferase reporter vector (Promega, Madison, Wisconsin, USA). A375 and SK-MEL-28 were co-transfected luciferase reporter vectors with miR-610 mimic or NC mimic for 48 h. Luciferase activities were determined using Dual-Luciferase^®^ Reporter Assay System (Promega).

### 2.9 Western blot

Cells were lysed in RIPA buffer (Beyotime) and then centrifuged at 12,000× *g* for 60 minutes to harvest supernatants. Protein samples in the supernatants were separated by sodium dodecyl-sulfate polyacrylamide gel electrophoresis (SDS-PAGE) and electrotransferred onto the PVDF membrane. The membrane was blocked with 5% skim milk and probed with primary antibodies: anti-vimentin and anti-snail (1:2,000), anti-N-cadherin and anti-E-cadherin (1:3,000), anti-NUCB2 and anti-GAPDH (1:4,000). Membranes were incubated with horseradish peroxidase-labeled secondary antibody (1:5,000) and subjected to enhanced chemiluminescence (KeyGen Biotech, Jiangsu, China) to detect immunoreactivities. All the antibodies were purchased from Santa Cruz Biotechnology (Santa Cruz, CA, USA).

### 2.10 Statistical analysis

All the data were expressed as mean ± SEM and analyzed via student’s *t*-test or one-way analysis of variance in GraphPad Prism software. *p* < 0.05 was considered statistically significant.


**Ethics approval:** This article does not involve any studies with human participants or animals performed by any of the authors.

## 3 Results

### 3.1 LINC00511-promoted cell proliferation of melanoma

**Figure 1:**
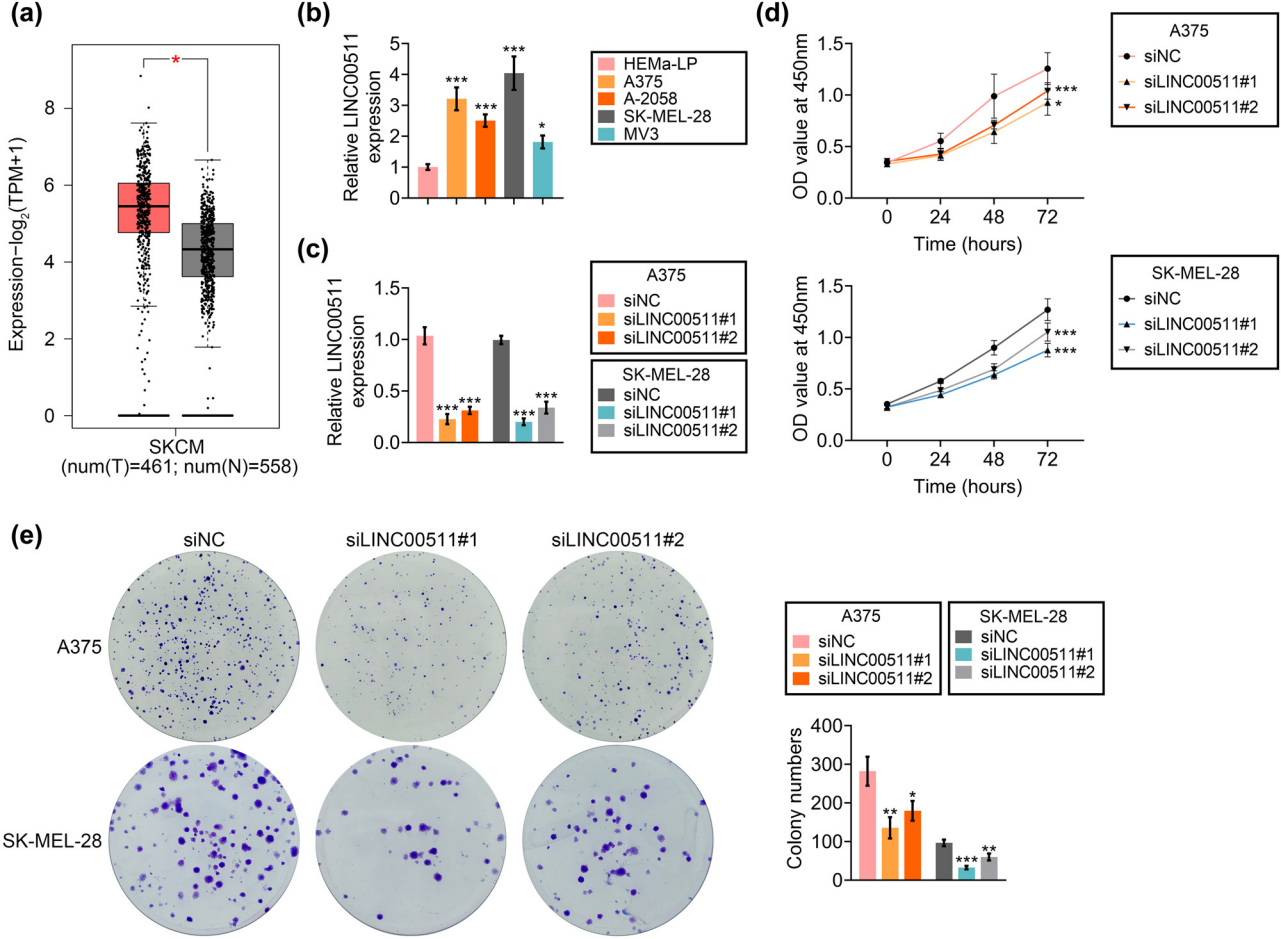
LINC00511-promoted Q3 cell proliferation of melanoma. (a) LINC00511 was increased in skin cutaneous melanoma (SKCM) tissues compared to normal tissues. (b) LINC00511 was also elevated in melanoma cells (A375, A-2058, SK-MEL-28, MV3) compared to HEMa-LP. (c) Transfection with si LINC00511#1 or si LINC00511#2 reduced expression of LINC00511 in A375 and SK-MEL-28. (d) Transfection with si LINC00511#1 or si LINC00511#2 decreased the cell viability of A375 and SK-MEL-28. (e) Transfection with siLINC00511#1 or si LINC00511#2 decreased cell proliferation of A375 and SK-MEL-28. *, **, *** vs normal tissues, HEMa-LP, or siNC, *p* < 0.05, *p* < 0.01, *p* < 0.001.

Bioinformatic analysis based on GEPIA showed that LINC00511 was increased in skin cutaneous melanoma (SKCM) tissues compared to normal tissues (Figure 1a). Furthermore, LINC00511 was also elevated in melanoma cells compared to HEMa-LP (Figure 1b). A375 and SK-MEL-28 were then transfected with si LINC00511#1 or si LINC00511#2 to reduce LINC00511 expression in melanoma cells (Figure 1c). Knockdown of LINC00511 decreased cell viability (Figure 1d) and inhibited proliferation (Figure 1e) of A375 and SK-MEL-28.

### 3.2 LINC00511-promoted cell metastasis of melanoma

**Figure 2:**
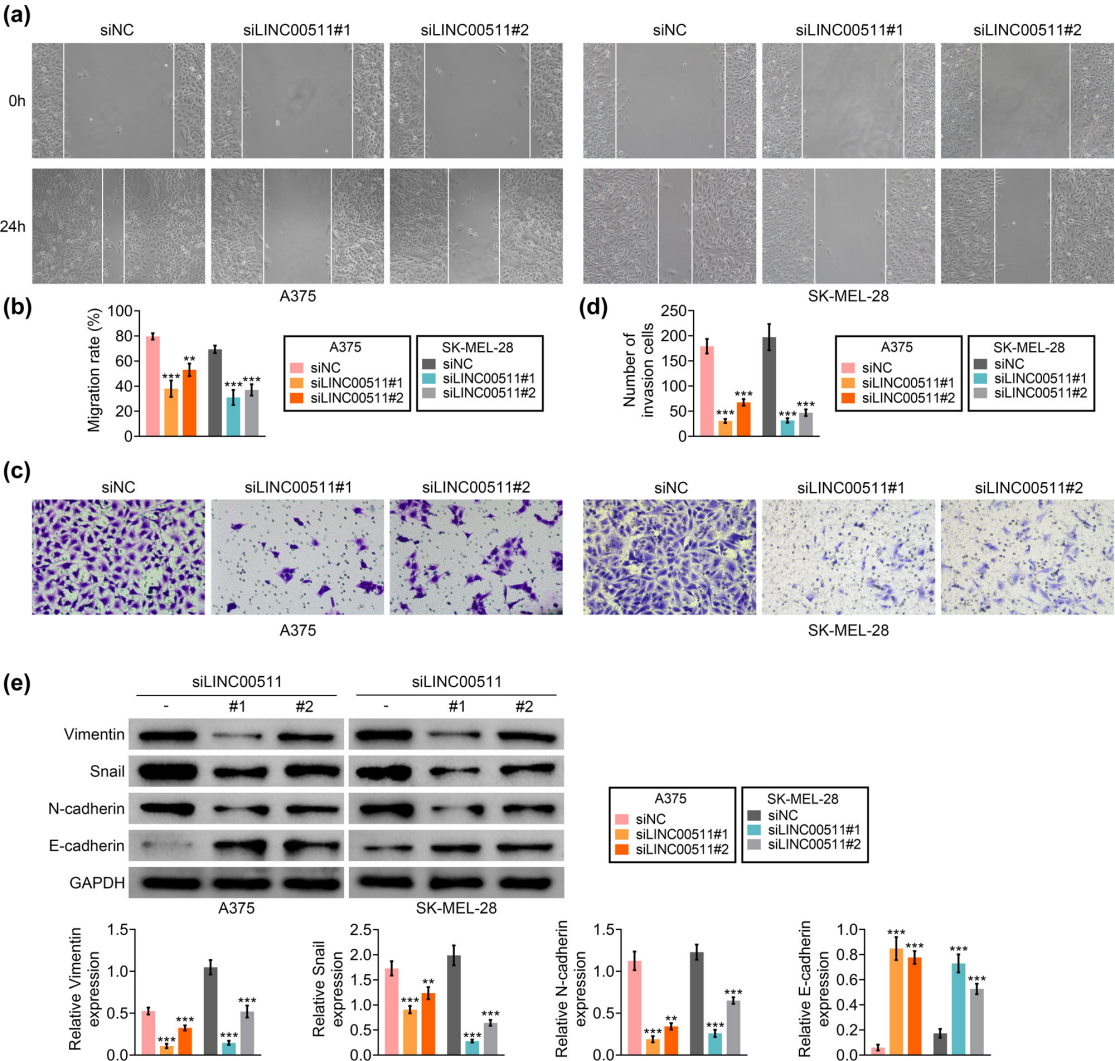
LINC00511-promoted cell metastasis of melanoma. (a) Transfection with si LINC00511#1 or si LINC00511#2 decreased cell migration of A375 and SK-MEL-28. (b) Relative migration cells in A375 and SK-MEL-28 that were transfected with si LINC00511#1 or si LINC00511#2. (c) Transfection with si LINC00511#1 or si LINC00511#2 decreased cell invasion of A375 and SK-MEL-28. (d) Relative invasion cells in A375 and SK-MEL-28 that were transfected with si LINC00511#1 or si LINC00511#2. (e) Transfection with si LINC00511#1 or si LINC00511#2 upregulated protein expression of E-cadherin, downregulated vimentin, snail, and N-cadherin in A375 and SK-MEL-28. **, *** vs siNC, *p* < 0.01, *p* < 0.001.

Cell migration of A375 and SK-MEL-28 was repressed by transfection with si LINC00511#1 or si LINC00511#2 (Figure 2a and b). Silence of LINC00511 also retarded cell invasion of A375 and SK-MEL-28 (Figure 2c and d). Loss of LINC00511 upregulated protein expression of E-cadherin, downregulated vimentin, snail, and N-cadherin in A375 and SK-MEL-28 (Figure 2e).

### 3.3 LINC00511 binds to miR-610

**Figure 3:**
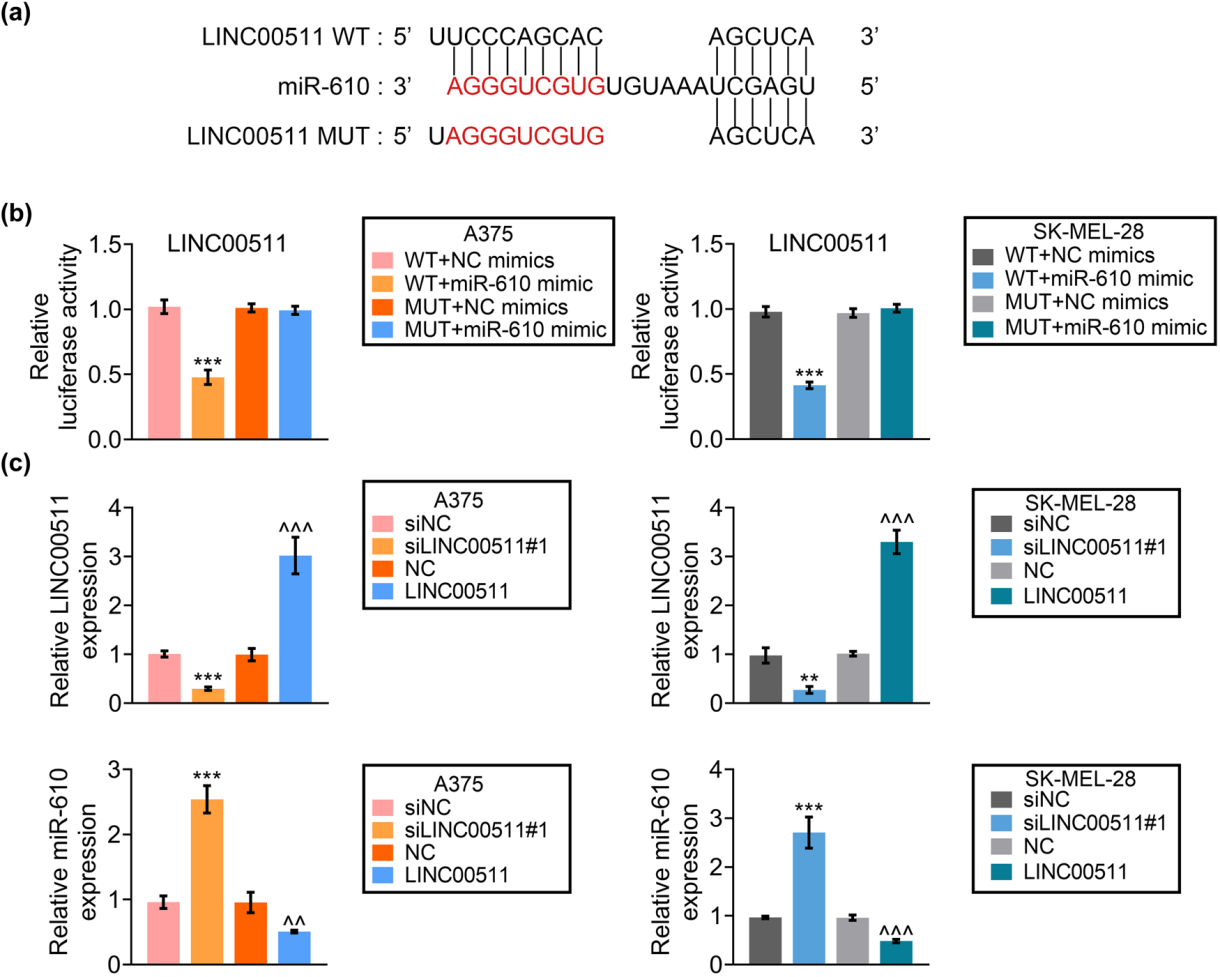
LINC00511 binds to miR-610. (a) Potential binding site between LINC00511 and miR-610 was predicted using BiBiServ. (b) Transfection with miR-610 mimics decreased luciferase activity of pmirGLO-WT-LINC00511 in A375 and SK-MEL-28. (c) Silence of LINC00511 decreased LINC00511 expression and increased miR-610 expression, while over-expression of LINC00511 increased LINC00511 and decreased miR-610 in A375 and SK-MEL-28. **, *** vs siNC, *p* < 0.01, *p* < 0.001. ^^, ^^^ vs NC, *p* < 0.01, *p* < 0.001.

Potential binding site between LINC00511 and miR-610 was predicted using BiBiServ (Figure 3a). Transfection with miR-610 mimics decreased luciferase activity of pmirGLO-WT-LINC00511 (Figure 3b). A375 and SK-MEL-28 were then transfected with si LINC00511#1 or pcDNA-LINC00511 to reduce or enhance LINC00511 expression in melanoma cells (Figure 3c). Silence of LINC00511 increased miR-610 expression, while over-expression of LINC00511 decreased miR-610 (Figure 3c).

### 3.4 miR-610 binds to NUCB2

**Figure 4:**
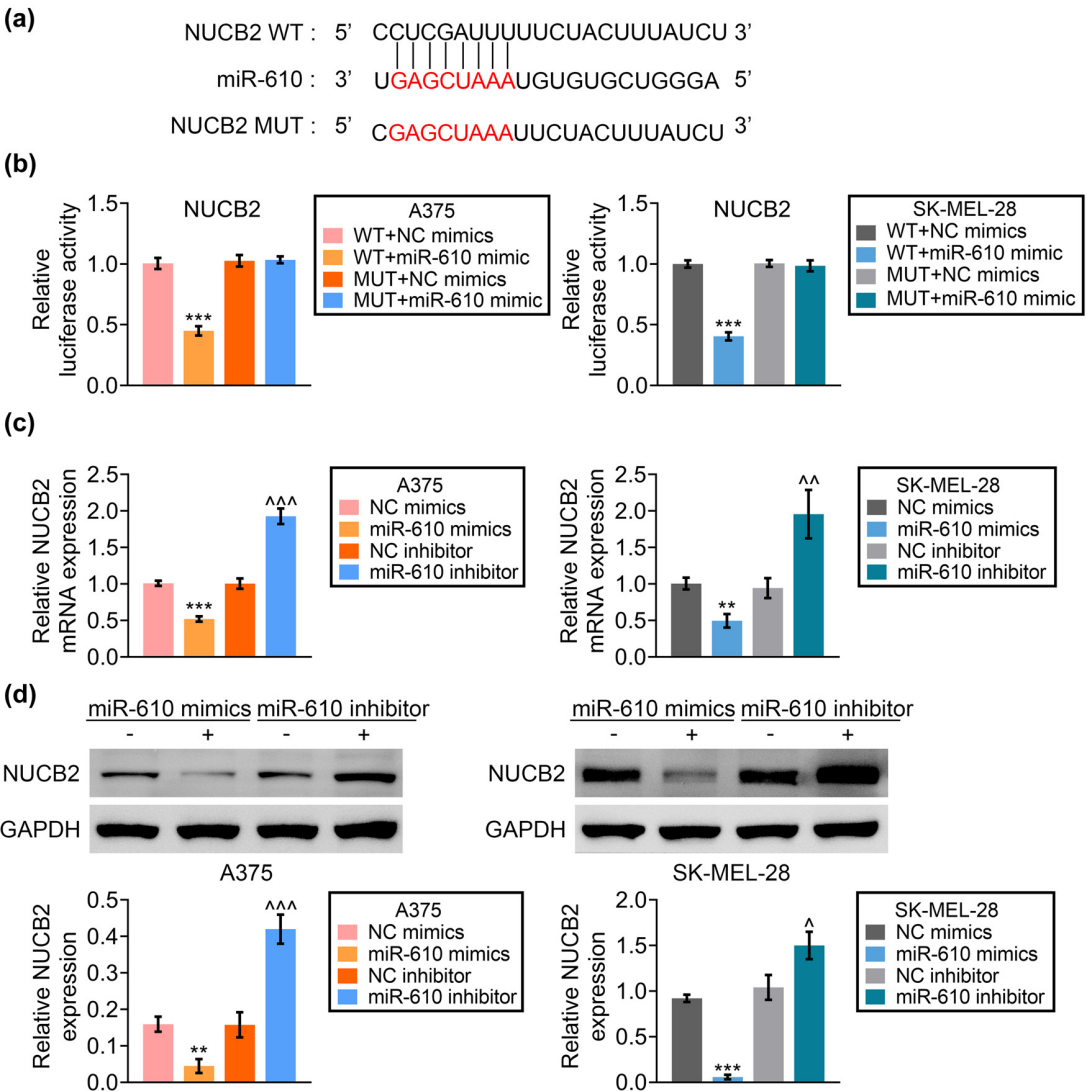
miR-610 binds to NUCB2. (a) Potential binding site between NUCB2 and miR-610 using miRDB. (b) Over-expression of miR-610 decreased the luciferase activity of pmirGLO-WT-NUCB2 in A375 and SK-MEL-28. (c) Silence of miR-610 increased mRNA expression of NUCB2, while over-expression of miR-610 decreased NUCB2 in A375 and SK-MEL-28. (d) Silence of miR-610 increased protein expression of NUCB2, while over-expression of miR-610 decreased NUCB2 in A375 and SK-MEL-28. **, *** vs siNC, *p* < 0.01, *p* < 0.001. ^, ^^, ^^^ vs NC, *p* < 0.05, *p* < 0.01, *p* < 0.001.

NUCB2 was predicted as the binding target of miR-610 using miRDB (http://mirdb.org/) (Figure 4a). Over-expression of miR-610 decreased the luciferase activity of pmirGLO-WT-NUCB2 (Figure 4b). A375 and SK-MEL-28 were then transfected with miR-610 mimic or inhibitor. Silence of miR-610 increased NUCB2 expression, while over-expression of miR-610 decreased NUCB2 (Figure 4c and d).

### 3.5 LINC00511-promoted melanoma progression through regulation of miR-610/NUCB2

**Figure 5:**
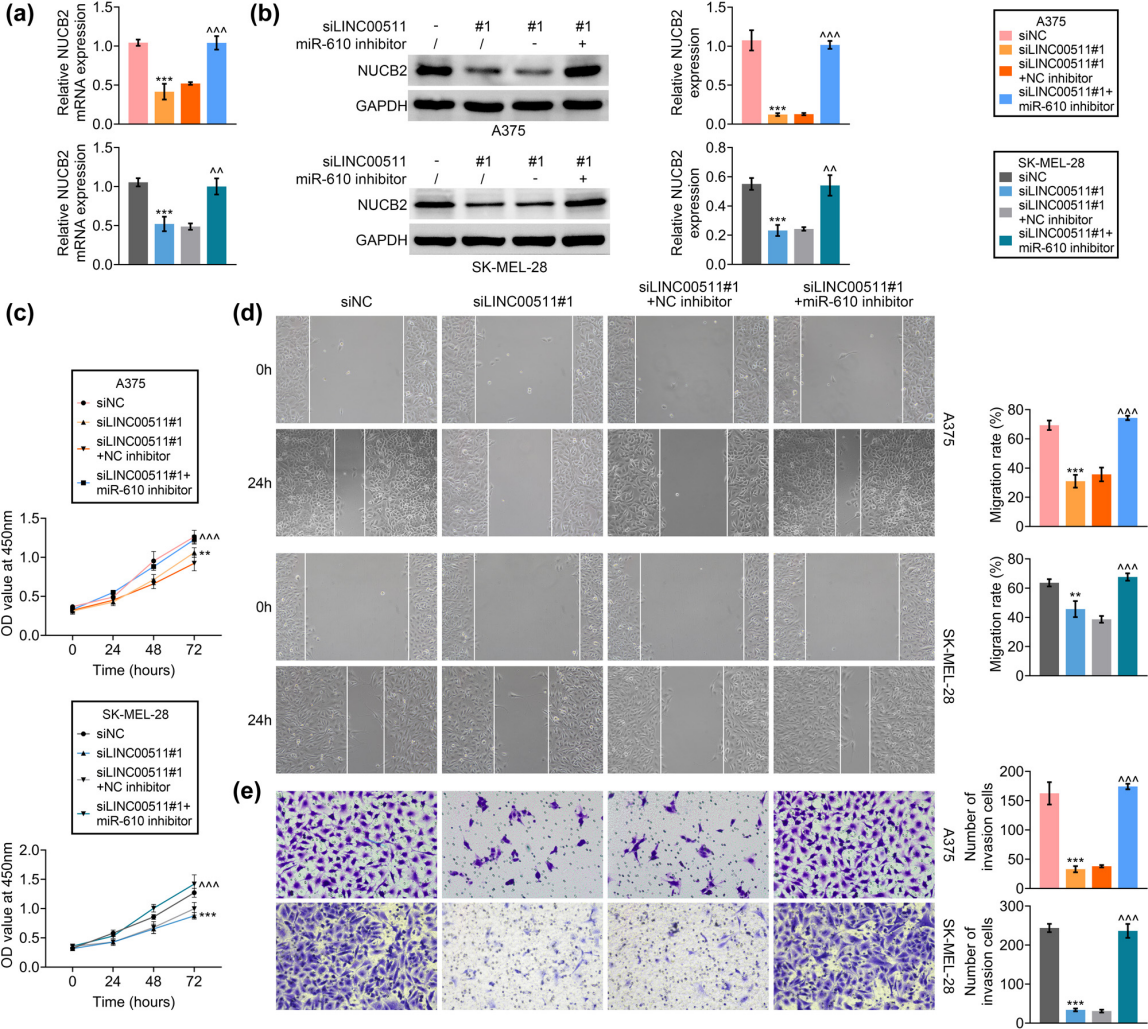
LINC00511-promoted melanoma progression through the regulation of miR-610/NUCB2. (a) Transfection with si LINC00511#1 reduced mRNA expression of NUCB2, while inhibition of miR-610 attenuated LINC00511 deficiency-induced decrease of NUCB2 in A375 and SK-MEL-28. (b) Transfection with si LINC00511#1 reduced protein expression of NUCB2, while inhibition of miR-610 attenuated LINC00511 deficiency-induced decrease of NUCB2 in A375 and SK-MEL-28. (c) Loss of miR-610 also weakened LINC00511 deficiency-induced decrease of cell viability in A375 and SK-MEL-28. (d) Loss of miR-610 also weakened the LINC00511 deficiency-induced decrease of cell migration in A375 and SK-MEL-28. (e) Loss of miR-610 also weakened the LINC00511 deficiency-induced decrease of cell invasion in A375 and SK-MEL-28. *** vs si LINC00511#1, *p* < 0.001. ^^, ^^^ vs NC, *p* < 0.01, *p* < 0.001.

A375 and SK-MEL-28 were cotransfected with si LINC00511#1 and miR-610 inhibitor. Transfection with si LINC00511#1 reduced expression of NUCB2 (Figure 5a and b), while inhibition of miR-610 attenuated LINC00511 deficiency-induced decrease of NUCB2 in A375 and SK-MEL-28 (Figure 5a and b). Loss of miR-610 also weakened LINC00511 deficiency-induced decrease of cell viability (Figure 5c), migration (Figure 5d), and invasion (Figure 5e) in A375 and SK-MEL-28.

## 4 Discussion

Splicing factor, proline- and glutamine-rich functioned as an oncogene in melanoma through interaction with LINC00511, and enrichment of LINC00511-promoted glycolysis of melanoma cells [13]. This study found that LINC00511 was an oncogenic lncRNA in melanoma, and loss of LINC00511 suppressed cell proliferation and metastasis of melanoma.

Previous research has shown that LINC00511 was enriched with splicing factor, proline- and glutamine-rich in melanoma cells [13]. Our results also identified the upregulation of LINC00511 in melanoma tissues and cells. Functional assays showed that the knockdown of LINC00511 decreased cell viability, reduced proliferation, invasion, and migration of melanoma cells. Epithelial–mesenchymal transition was associated with prometastatic and invasive phenotypes of melanoma [17]. Inhibition of LINC00511 increased epithelial biomarker (E-cadherin) expression and suppressed mesenchymal biomarkers (vimentin, snail, N-cadherin) to inhibit epithelial–mesenchymal transition of lung cancer [18]. In this study, loss of LINC00511 also upregulated E-cadherin expression, downregulated Vimentin, snail, and N-cadherin to suppress epithelial–mesenchymal transition of melanoma.

MiRNAs were related to cell proliferation, metastasis, and drug resistance of melanoma through the regulation of downstream mRNAs [19]. LncRNAs–miRNAs–mRNAs network mediated melanoma progression [14]. MiR-625-5p was the target of LINC00511 in melanoma cells, and depletion of LINC00511 enhanced the transcript level of miR-625-5p and inhibited glycolysis [13]. MiR-610, as a tumor suppressor in gastric cancer [20], glioblastoma [21], and melanoma [16], was identified as a target of LINC00511 in melanoma cells. Silence of LINC00511 increased miR-610 in melanoma cells, and inhibition of miR-610 attenuated LINC00511 deficiency-induced decrease of cell viability, migration, and invasion of melanoma, suggesting that LINC00511 might contribute to melanoma progression through downregulation of miR-610.

Furthermore, our results demonstrated that NUCB2 was a target gene of miR-610. NUCB2 functions as a DNA/Ca^2+^ -binding protein and participates in the regulation of immune system, glucose metabolism, and food intake [22]. NUCB2 exerted either pro-metastatic or anti-metastatic in distinct tumors through modulation of various signalings [23]. Endoplasmic reticulum stress stimulated the expression of Krüppel-like factor 4, and Krüppel-like factor 4 binds to promoter region and facilitated transcription of NUCB2 [24]. Upregulation of NUCB2 was associated with the inhibition of apoptosis and promotion of cell metastasis in melanoma cells [24]. Silence of LINC00511 reduced expression of NUCB2 in melanoma cells, and inhibition of miR-610 attenuated LINC00511 deficiency-induced decrease of NUCB2. Therefore, LINC00511 might contribute to melanoma progression through upregulation of miR-610-mediated NUCB2. However, whether over-expression of NUCB2 could reverse the suppressive effect of LINC00511 deficiency on melanoma progression should be investigated in further research.

Collectively, LINC00511 was an oncogene in melanoma. Loss of LINC00511 inhibited proliferation, metastasis, and epithelial–mesenchymal transition of melanoma through the increase of miR-610 and the decrease of NUCB2. Therefore, LINC00511/miR-610/NUCB2 was a potential target of melanoma. However, the effect of LINC00511 on *in vivo* tumor growth of melanoma should be investigated in further research.
